# Predicting disease‐specific survival in patients undergoing active surveillance for papillary thyroid carcinoma

**DOI:** 10.1002/wjs.12434

**Published:** 2024-12-19

**Authors:** Stanton Nielsen, Kristine Kuchta, Grace Huang, Samuel Zuber, Simon Holoubek, Amanda Karcioglu, Amna Khokar, Richard Prinz, Tricia Moo‐Young

**Affiliations:** ^1^ Department of Surgery NorthShore University HealthSystem Evanston IL USA; ^2^ The University of Chicago Medical Center Chicago Chicago IL USA; ^3^ Department of Surgery University of Wisconsin‐Madison Madison WI USA; ^4^ Department of Surgery John H. Stroger Jr, Hospital of Cook County Chicago IL USA

**Keywords:** endocrine, head and neck, oncology, thyroid

## Abstract

**Background:**

American Thyroid Association guidelines support active surveillance (AS) for low‐risk papillary thyroid cancer (PTC). We developed a calculator to aid patient selection.

**Methods:**

From 2004 to 2020, 148,904 PTC patients were selected from the surveillance, epidemiology, and end results (SEER) database. Univariable and multivariable analysis evaluated patient and treatment characteristics. Patients were randomly allocated into training (80%) or validation sets (20%). Coefficients generated a mathematical model to predict 5‐ and 10‐year disease‐specific survival (DSS).

**Results:**

The mean DSS was 15.5 years with a 5‐ and 10‐year DSS of 99.3% and 98.6%, respectively. Age, sex, race, median household income (MHI), tumor size, and nodal status were significant on multivariable analysis (*p* ≤ 0.05) and included variables in our calculator. 2404 patients underwent non‐operative management (NOM) and were more likely older, male, higher MHI, larger tumor size, and less nodal positivity. Area under the curve (AUC) for 5‐ and 10‐year DSS were 0.83 and 0.81, respectively, for the training set and 0.81 and 0.79, respectively, for the validation set.

**Example:**

65‐year‐old White female with a 0.8 cm PTC, cN0 with a MHI ≥ $75,000, had a 10‐year predicted DSS was 95.6% with NOM and 99.3% with surgery. Alternatively, changing the patient's race to Hispanic, the 10‐year predicted DSS was 94.1% with NOM and 99.0% with surgery.

**Conclusions:**

As awareness of AS for PTC expands, it is important to consider objective data to guide informed decision making. This validated calculator is a useful tool to predict DSS for patients considering AS for PTC.

## 1 INTRODUCTION

Papillary thyroid carcinoma (PTC) is the most common endocrine malignancy, and its incidence has increased in recent decades.^1^, ^2^, ^3^ Its rising incidence is attributed to incidental findings on diagnostic imaging and detection of smaller tumors.^4^, ^5^, ^6^, ^7^ Despite its rise, mortality from PTC is relatively unchanged.^2^ As growth kinetics of PTC have been unveiled, there have been increased efforts to tailor and decrease the morbidity of treatment regimens. Active surveillance (AS) for low‐risk papillary thyroid microcarcinoma (PTMC) is helping in this effort.^8^, ^9^ Early studies on AS showed similar survival outcomes for PTMC patients treated with operative and non‐operative management (NOM).^8^, ^9^, ^10^, ^11^ Others have investigated the use of AS for tumors >1 cm and found similar favorable outcomes to AS for PTMC.^12^, ^13^, ^14^, ^15^ In 2015, AS was included as a treatment option for PTC in the American Thyroid Association (ATA) guidelines.^16^

As adoption of AS increases, it is important to consider the diversity of clinical and pathologic variables a patient may present with at the time of diagnosis. Additionally, many of the prior studies on AS have been conducted in non‐United States (US) populations. Our primary objective is to develop a validated risk assessment calculator for predicting treatment outcomes in patients with PTC while secondarily identifying variables beyond size that influence disease‐specific survival (DSS). This tool could aid in patient selection for AS while considering a variety of patient and disease specific factors.

## 2 METHODS

### 2.1 Data source and patient selection

**FIGURE 1:**
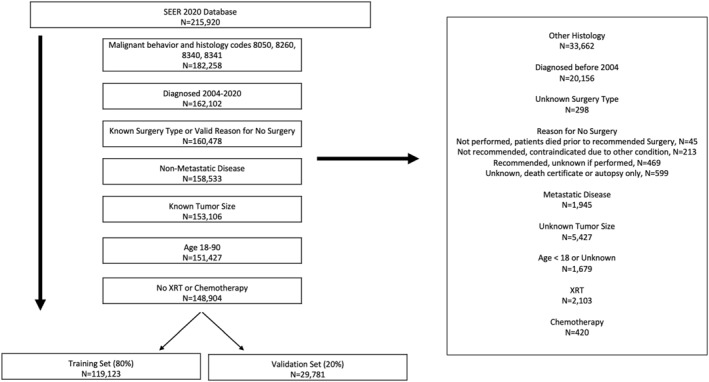
Patient selection criteria.

IRB approval was not required for this study. The SEER database (SEER*STAT version 8.4.2) was queried for PTC patients between 2004 and 2020. Histology codes were included from International Classification of Diseases (ICD O‐3): 8050 (PTC NOS), 8260 (PTC), 8340 (PTC follicular variant), 8341 (PTMC). Adult patients, >18‐years‐old, with known surgery type or with valid reasons for NOM (Figure 1) were included. Patients with distant metastasis, unknown tumor size, and recipients of adjuvant treatments were excluded. Patient variables included: age, sex, race, median household income (MHI), tumor size, and clinical nodal staging (Table 1). Patient age was divided into decades: <30, 30–39, 40–49, 50–59, 60–69, ≥70‐years‐old. Tumor sizes were categorized by centimeters (cm): 0–0.5 cm, 0.6–1.0 cm, 1.1–1.5 cm, 1.6–2.0 cm, 2.1–4 cm, and >4 cm. Nodal status was based on clinical nodal stages cN0, cN1, and cNx.

**TABLE 1:** Characteristics of patients.

	All	Training	Validation	*p*‐value
	N (%)	N (%)	N (%)	‐
Total patients	148,904	119,123	29,781	‐
Year of diagnosis				0.311
2004	5351 (3.6)	4278 (3.6)	1073 (3.6)	
2005	5899 (4.0)	4702 (3.9)	1197 (4.0)	
2006	6392 (4.3)	5157 (4.3)	1235 (4.1)	
2007	7027 (4.7)	5603 (4.7)	1424 (4.8)	
2008	7904 (5.3)	6334 (5.3)	1570 (5.3)	
2009	8705 (5.8)	7029 (5.9)	1676 (5.6)	
2010	8831 (5.9)	7059 (5.9)	1772 (6.0)	
2011	9505 (6.4)	7585 (6.4)	1920 (6.4)	
2012	9869 (6.6)	7936 (6.7)	1933 (6.5)	
2013	10,297 (6.9)	8210 (6.9)	2087 (7.0)	
2014	10,373 (7.0)	8270 (6.9)	2103 (7.1)	
2015	10,701 (7.2)	8478 (7.1)	2223 (7.5)	
2016	10,260 (6.9)	8161 (6.9)	2099 (7.0)	
2017	9802 (6.6)	7855 (6.6)	1947 (6.5)	
2018	9994 (6.7)	8067 (6.8)	1927 (6.5)	
2019	9863 (6.6)	7927 (6.7)	1936 (6.5)	
2020	8131 (5.5)	6472 (5.4)	1659 (5.6)	
Age, years [mean ± SD]	50 ± 15	50 ± 15	50 ± 15	0.976
Age, years				0.270
< 30	14,296 (9.6)	11,479 (9.6)	2817 (9.5)	
30–39	26,559 (17.8)	21,201 (17.8)	5358 (18.0)	
40–49	33,429 (22.5)	26,735 (22.4)	6694 (22.5)	
50–59	34,743 (23.3)	27,871 (23.4)	6872 (23.1)	
60–69	24,967 (16.8)	19,862 (16.7)	5105 (17.1)	
≥ 70	14,910 (10.0)	11,975 (10.1)	2935 (9.9)	
Sex				0.221
Male	34,192 (23.0)	27,433 (23.0)	6759 (22.7)	
Female	114,712 (77.0)	91,690 (77.0)	23,022 (77.3)	
Race				0.351
White	95,355 (64.0)	76,422 (64.2)	18,933 (63.6)	
Black	8612 (5.8)	6847 (5.7)	1765 (5.9)	
Hispanic	26,044 (17.5)	20,748 (17.4)	5296 (17.8)	
Asian/Pacific Islander	16,743 (11.2)	13,385 (11.2)	3358 (11.3)	
Other	2150 (1.4)	1721 (1.4)	429 (1.4)	
Median household income				0.454
< $60,000	29,936 (20.1)	23,889 (20.1)	6047 (20.3)	
$60,000–74,999	52,758 (35.4)	42,288 (35.5)	10,470 (35.2)	
≥ $75,000	66,210 (44.5)	52,946 (44.4)	13,264 (44.5)	
Sequence number				0.377
First and only cancer	119,518 (80.3)	95,560 (80.2)	23,958 (80.4)	
Multiple primary cancers	29,386 (19.7)	23,563 (19.8)	5823 (19.6)	
Tumor size, cm				0.335
0–0.5	31,788 (21.3)	25,325 (21.3)	6463 (21.7)	
0.6–1.0	29,662 (19.9)	23,751 (19.9)	5911 (19.8)	
1.1–1.5	27,718 (18.6)	22,195 (18.6)	5523 (18.5)	
1.6–2.0	17,306 (11.6)	13,795 (11.6)	3511 (11.8)	
2.1–4.0	31,641 (21.2)	25,424 (21.3)	6217 (20.9)	
> 4.0	10,789 (7.2)	8633 (7.2)	2156 (7.2)	
N Stage				0.093
N0	108,570 (72.9)	87,005 (73.0)	21,565 (72.4)	
N1	35,103 (23.6)	27,956 (23.5)	7147 (24.0)	
NX	5231 (3.5)	4162 (3.5)	1069 (3.6)	
Surgery				0.133
Active surveillance	2404 (1.6)	1930 (1.6)	474 (1.6)	
Lobectomy	23,831 (16.0)	18,952 (15.9)	4879 (16.4)	
Total thyroidectomy	122,669 (82.4)	98,241 (82.5)	24,428 (82.0)	

### 2.2 Statistical analysis

**FIGURE 2:**
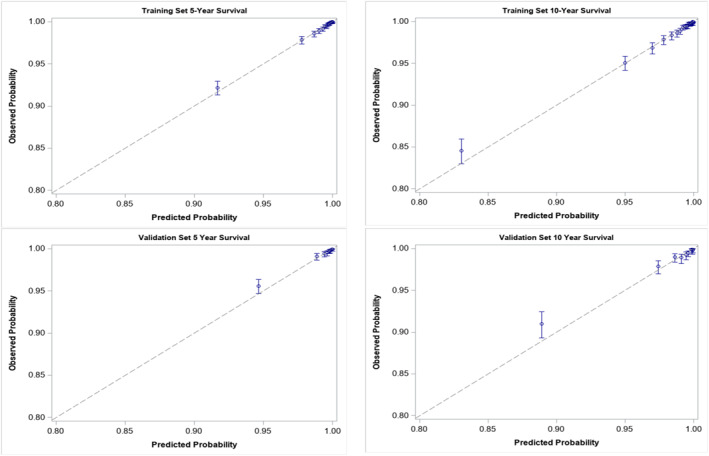
Calibration plots for 5‐ and 10‐year disease specific survival in training and validation cohorts.

Patients were randomly allocated into training (80%) or validation (20%) sets. Comparisons were made between sets using chi‐square and independent sample *t*‐tests. Univariable and multivariable analysis were performed using Cox regression (Table 2), evaluating the impact of patient and treatment characteristics on DSS. Hazard ratios (HR) with confidence intervals of 95% were reported and used to identify variables to include in the clinical calculator. Baseline 5‐ and 10‐year rates of DSS were calculated for the Cox regression model using the PHREG procedure and the BASELINE function in SAS 9.4 (SAS Institute, Cary, NC). This baseline corresponded to estimated survival rates when all model covariates were set to the reference group. Final model parameters were obtained using 100 bootstrap samples, providing a sample size that attained a normal distribution of the coefficients, with the mean value chosen as the final coefficient. Calibration plots, receiver operating characteristic (ROC) curve analysis, and area under the ROC curve (AUC) were used to assess the model's performance. The final model was applied to the validation cohort, using calibration plots, ROC curves, and AUC to assess model performance in the training and validation cohort (Figure 2). Calibration plots were created by splitting patients into risk vigintiles (Supplemental Table 1) for the training cohort and risk deciles (Supplemental Table 2) for the validation cohort. We calculated, observed and predicted 5‐ and 10‐year DSS for each group using Kaplan‐Meier methods. All statistical analysis was performed using SAS 9.4 (SAS Institute, Cary, NC) and statistical significance set at *p* < 0.05.

**TABLE 2:** Univariable and multivariable survival analysis.

	Univariable		Multivariable	
	Hazard ratio (95% CI)	*p*‐value	Hazard ratio (95% CI)	*p*‐value
Age, years				
< 30	Reference	‐	Reference	‐
30–39	1.54 (0.65–3.65)	0.324	2.05 (0.87–4.85)	0.102
40–49	5.97 (2.77–12.83)	**<0.001**	9.33 (4.34–20.08)	**<0.001**
50–59	14.44 (6.81–30.62)	**<0.001**	25.26 (11.91–53.61)	**<0.001**
60–69	32.07 (15.18–67.78)	**<0.001**	54.73 (25.86–115.82)	**<0.001**
≥ 70	91.86 (43.57–193.67)	**<0.001**	137.72 (65.22–290.81)	**<0.001**
Sex				
Male	2.55 (2.28–2.85)	**<0.001**	1.35 (1.20–1.52)	**<0.001**
Female	Reference	‐	Reference	‐
Race				
White	Reference	‐	Reference	‐
Black	1.25 (1.00–1.57)	0.053	1.34 (1.06–1.68)	**0.013**
Hispanic	1.27 (1.10–1.47)	**0.001**	1.36 (1.17–1.58)	**<0.001**
Asian/Pacific Islander	1.05 (0.87–1.26)	0.605	1.04 (0.86–1.26)	0.672
Other	0.41 (0.18–0.91)	**0.028**	0.53 (0.24–1.18)	0.119
Median household income				
< $60,000	1.32 (1.14–1.54)	**<0.001**	1.36 (1.17–1.59)	**<0.001**
$60,000–74,999	1.38 (1.21–1.56)	**<0.001**	1.31 (1.15–1.48)	**<0.001**
≥ $75,000	Reference	‐	Reference	‐
Tumor size, cm				
0–0.5	Reference	‐	Reference	‐
0.6–1.0	1.05 (0.82–1.35)	0.714	1.16 (0.90–1.49)	0.247
1.1–1.5	1.13 (0.88–1.45)	0.355	1.16 (0.90–1.50)	0.249
1.6–2.0	1.68 (1.30–2.16)	**<0.001**	1.65 (1.27–2.14)	**<0.001**
2.1–4.0	3.27 (2.68–4.00)	**<0.001**	3.04 (2.46–3.74)	**<0.001**
>4.0	10.07 (8.23–12.32)	**<0.001**	7.77 (6.29–9.60)	**<0.001**
N Stage				
N0	Reference	‐	Reference	‐
N1	2.95 (2.64–3.31)	**<0.001**	3.18 (2.81–3.59)	**<0.001**
NX	2.35 (1.74–3.19)	**<0.001**	1.31 (0.96–1.78)	0.088
Surgery				
Active surveillance	9.15 (7.51–11.14)	**<0.001**	6.06 (4.94–7.44)	**<0.001**
Lobectomy	0.75 (0.63–0.90)	**0.002**	1.22 (1.00–1.47)	**0.046**
Total thyroidectomy	Reference	‐	Reference	‐

*Note*: Bold values were found to be statistically significant.

## 3 RESULTS

### 3.1 Patient and tumor characteristics

A total of 148,904 patients were identified using our inclusion and exclusion criteria. No differences were observed between the training and validation sets (Table 1). There were 146,500 surgical and 2404 NOM patients. At the later part of the study, an increasing percentage of patients were managed with NOM compared to the earlier period. The mean percentage of patients undergoing NOM was 1.27% annually prior to 2015 versus 2.08% following 2015. The mean DSS for the entire cohort was 15.5 years with a 5‐ and 10‐year DSS of 99.3% and 98.6%, respectively. Operative patients had a mean DSS of 15.5 ± 0.0 years with a 5‐ and 10‐year DSS of 99.4% and 98.7% respectively. NOM had a mean DSS of 14.4 ± 0.1 years with a 5‐ and 10‐year DSS of 92.8% and 91.0%, respectively.

### 3.2 Multivariable analysis of pathologic features

When NOM patients were compared to operative patients, NOM patients were on average more likely to be older (56 ± 18 years vs. 50 ± 15 years), male (33.2% male vs. 22.9%, *p* < 0.0001), and had a higher MHI (>$60,000 85.8% vs. 79.9%, *p* < 0.0001). Median tumor size was 1.6 cm for NOM compared to 1.3 cm in operative patients (*p* < 0.0001). There was less nodal positivity among NOM patients (*N* ^1^ = 13.6%) versus operative patients (*N* ^1^ = 23.6%), *p*‐value <0.0001 (Supplemental Table 3). Patient treatment factors that were statistically significant (*p* < 0.05) on univariable and multivariable analysis were age, sex, race, MHI, tumor size, and nodal status (Table 2). The final prediction model utilized HR to predict DSS for each variable (Table 3). With each decade increase in age the HR doubled. Increasing tumor size demonstrated an upward trend in HR, where tumors >4 cm had seven times higher risk of disease specific mortality compared to tumors <0.5 cm (HR = 7.48). Male patients were associated with a HR of 1.35 compared to female patients. Black and Hispanic patients had the highest HR of 1.34 and 1.36, respectively, compared to white patients. Having an annual MHI <$60,000 had the highest risk of mortality (HR 1.36). Nodal positivity increased disease specific mortality more than three times that of node negative patients (HR 3.11).

**TABLE 3:** Final prediction model.

	Coefficient	SE	HR (95% CI)
Age, years			
< 30	0	‐	Reference variable
30–39	0.72	0.44	2.05 (0.87–4.84)
40–49	2.23	0.39	9.32 (4.33–20.05)
50–59	3.23	0.38	25.20 (11.87–53.47)
60–69	4.00	0.38	54.62 (25.81–115.58)
≥ 70	4.93	0.38	137.83 (65.27–291.06)
Sex			
Male	0.30	0.06	1.35 (1.20–1.52)
Female	0	‐	Reference variable
Race			
White	0	‐	Reference
Black	0.29	0.12	1.34 (1.07–1.68)
Hispanic	0.31	0.08	1.36 (1.17–1.58)
Asian/Pacific Islander	0.04	0.10	1.04 (0.86–1.26)
Other	−0.63	0.41	0.53 (0.24–1.18)
Median household income			
< $60,000	0.31	0.08	1.36 (1.17–1.59)
$60,000–74,999	0.27	0.07	1.30 (1.15–1.48)
≥ $75,000	0	‐	Reference
Tumor size, cm			
0–0.5	0	‐	Reference
0.6–1.0	0.12	0.13	1.13 (0.88–1.45)
1.1–1.5	0.11	0.13	1.12 (0.87–1.44)
1.6–2.0	0.46	0.13	1.59 (1.23–2.05)
2.1–4.0	1.07	0.10	2.92 (2.38–3.59)
> 4.0	2.01	0.11	7.48 (6.08–9.21)
N Stage			
N0	0	‐	Reference
N1	1.13	0.06	3.11 (2.76–3.50)
NX	0.27	0.16	1.31 (0.96–1.79)
Surgery			
Active surveillance	1.78	0.10	5.93 (4.83–7.26)
Surgery	0	‐	Reference

The final prediction model utilized bootstrapped coefficients (Table 3) for each of the significant patient and tumor characteristics. The AUC values for the training set were 0.83 and 0.81, respectively for 5‐ and 10‐year DSS, respectively. In the validation set, the AUC values were 0.81 and 0.79 for 5‐ and 10‐year DSS, respectively.

### 3.3 Utilization of calculator

We compared three scenarios to model varying clinical presentations of PTC (Table 4). In Scenario #1 a 57‐year‐old Hispanic female with a 0.8 cm PTC, cN0 and MHI ≥$75,000 has a predicted 10‐year DSS of 97.4% with NOM versus 99.5% with surgery. When the same patient presents with a 1.7 cm tumor the 10‐year DSS is similar with 96.1% from NOM and 99.3% with surgery. Scenario #2 describes a 65‐year‐old White female with a 0.8 cm PTC, cN0, and MHI ≥ $75,000. Her predicted 10‐year DSS is 95.6% with NOM and 99.3% with surgery. However, changing race for this patient to Hispanic, the predicted DSS at 10‐year is 94.1% with NOM versus 99.0% with surgery. In scenario #3, a 45‐year‐old Asian female presenting with a 1.2 cm PTC, cN0, and a MHI ≥ $75,000 has a predicted 10‐year DSS of 99.2% with NOM versus 99.9% with surgery. While changing age the for this patient to 65‐years‐old, we observed a predicted 10‐year DSS of 95.5% with NOM versus 99.2% with surgery.

**TABLE 4:** 10‐Year DSS (percentage).

	Tumor size (cm)	Age (years)	Sex	Race	Surgery	Active surveillance
Scenario #1	0.8	57	Female	Hispanic	99.5	97.4
	1.7				99.3	96.1
Scenario #2	0.8	65	Female	White	99.3	95.6
				Hispanic	99.0	94.1
Scenario #3	1.2	45	Female	Asian	99.9	99.2
		65			99.2	95.5

## 4 DISCUSSION

A landmark study from Kuma Hospital in Japan published outcomes of 162 patients successfully managed with AS.^8^ With eight years of follow up, >70% of these PTMC patients managed by AS had no baseline change in tumor size. A 2023 follow‐up study included 3222 patients managed by AS, only 6.6% of these patients had tumor enlargement ≥3 mm and all were successfully managed with conversion surgery.^17^ Several groups have reported similarly low rates of disease progression and successful outcomes with AS.^10^, ^11^, ^18^, ^19^, ^20^, ^21^ Slow rates of disease progression for PTMC have long been suggested based on autopsy series.^22^, ^23^ Collectively these findings support that PTMC represents an indolent tumor for which immediate surgical intervention is likely unnecessary.

Contemporary thyroid cancer guidelines and consensus statements consider AS a treatment option for select PTC patients.^16^, ^24^ In the later part of our study, there was an increased percentage of patients who underwent AS compared to those diagnosed earlier in the study. The steady increase in patients undergoing AS were in the years following 2015. This could represent early adoption of ATA guidelines published around that time. Although guidelines are meant as suggestions, not standards, it is important that as AS is adopted, its safety be examined across a spectrum of clinical settings. Our study examined a contemporary national cancer registry of >140,000 clinically diverse patients diagnosed with PTC within the US. The large volume and diverse nature of our patients allowed us to identify multiple variables that influenced DSS in patients with PTC.

Successful implementation of AS requires careful patient selection, routine ultrasound follow‐up, and care from experienced cancer teams. The NOM group in our study were patients for whom surgery was offered but declined or otherwise not recommended as first‐line by the treating provider. Prior studies have also used NOM patients in cancer registries as a surrogate for AS.^15^, ^25^ One such example was Ho et al. who studied AS in the SEER registry (1975–2015) and reported decreased survival outcomes for NOM patients based on tumor size and patient age.^15^

The landmark study by Ito et al. implemented AS for tumors <1.0 cm.^8^, ^15^ Tuttle et al. demonstrated tumor sizes up to 1.5 cm had similar growth kinetics to those <1.0 cm, arguing AS could be offered for tumors up to 1.5 cm.^18^ In our study, the HR for tumors >2.0 cm nearly doubled when compared to tumors <2.0 cm suggesting tumors up to 2.0 cm could be considered for AS. Ho et al. demonstrated no difference in survival outcomes between AS and surgical patients for tumors up to 2.0 cm.^14^ We explored increasing tumor size in our clinical scenarios and showed acceptable DSS for patients managed with AS or immediate surgery (Table 4). With our calculator's ability to alter tumor size as well as other clinical characteristics, a treating provider could inform patients on the associated risk of pursuing AS versus surgical intervention.

When age was examined in our calculator, a near doubling of the HR was seen with each decade of life. Age has long been a predictor of survival for PTC as seen in staging of differentiated thyroid cancer. The influence of age on AS outcomes has been explored by others.^18^, ^26^ In these studies, patients <50 years of age had faster rates of tumor growth compared to those >50 years of age. Despite having less favorable tumor growth kinetics, young patients maintain excellent survival outcomes with AS. Good survival outcomes are maintained in older patients treated with AS, but demonstrate a sharper decline for patients age >72 years.^15^ These studies along with our findings support a more tailored approach using AS beyond just tumor size.

Race and sex had a significant influence on DSS in our AS and surgery patients. This was most notable in Hispanic and Black patients. Using our validated calculator, we illustrated how a Hispanic patient had a lower 10‐year predicted DSS than a White patient presenting with otherwise identical characteristics. In our calculator male patients also had a decreased predicted DSS for AS when compared to female patients. The reason for these differences in DSS by race and sex is unclear. Differences in disease presentation and clinical behavior of PTC based on race and sex have been reported.^27^, ^28^, ^29^, ^30^ Findings in these studies suggest tumor biology may account for these differences, similar to how growth kinetics of PTC are influenced by age.^26^

Lastly, our study identified MHI as an independent predictor of DSS in patients with PTC. Patients with a MHI < $60,000 annully had worse outcomes compared to patients with a higher MHI. If applied in our calculator, a lower MHI would predict a decreased DSS for patients with otherwise identical clinical characteristics. In other cancers the effect of MHI on oncologic outcomes has been well documented.^31^, ^32^, ^33^, ^34^ Reasons for these disparities suggested by these studies include access to quality healthcare, financial distress from cost, lost wages related to cancer care, and possibly the dysregulation of stress pathways promoting aggressive tumor biology. While we do not believe our study supports withholding the option of AS from patients of a lower MHI, it does underpin the importance of providing resource support for patients with strained financial resources who are enrolled in an AS program.

Despite increasing evidence that AS is a safe form of management for select patients with PTC, there remain barriers to wide adoption of AS. One study reported 76% of physicians surveyed believed AS was an appropriate line of treatment, however, only 44% utilized it in their practice.^35^ Barriers for not implementing AS identified by the authors included patient refusal, concern of incomplete follow‐up, and provider concerns of misclassifying a patient's risk. Shared decision making is an important part of surgical planning. Avoiding implicit biases around a patient's ability to participate in an AS program should be strongly avoided. We believe our novel clinical prediction model could aid providers in having a patient centered discussion about AS options.

Clinical risk assessment calculators have been shown to enhance treatment discussions and shared decision making between physicians and patients.^36^ Previous studies demonstrate the benefit of clinical calculators in thyroid cancer.^37^, ^38^, ^39^ A clinical nomogram for AS was published out of China and identified risk factors that predicted a more aggressive clinical course in PTMC.^40^ Our calculator was derived from the US‐based SEER‐21 cancer registry, which accounts for approximately 35% of the US population.^41^ This expands upon the prior risk‐stratification framework by including age, race, sex, tumor size, nodal status and MHI when considering AS.

Our study has its limitations. Large databases have the potential for recording and coding errors. Additionally, it lacks variables such as tumor growth and use of molecular testing. The SEER database only records surgical intervention for patients who undergo surgery within the first year of diagnosis. Therefore, our NOM cohort may have subsequently undergone conversion to surgery.^42^ Our study analyzed DSS, subjecting it to possible attribution bias, seen in other disease processes, like prostate cancer.^43^ Our NOM group is a surrogate for AS and thus it is unknown whether they had routine ultrasounds or clinical follow up. Lastly, MHI in this US‐based cohort impacted DSS, but this may not be generalizable to countries with differing healthcare systems. Although this calculator may be a useful tool, it should not replace individualized care and an experienced multidisciplinary team.

## 5 CONCLUSION

This study evaluates a large, heterogenous PTC population and seeks to expand variables that can influence decision making around active surveillance. We developed a validated clinical calculator that provides predicted DSS for patients considering AS versus surgery. In this era of personalized medicine, utilization of a multi‐variable calculator could aid in shared decision making between patients and providers and allow for a more tailored approach in treating patients with PTC.

## AUTHOR CONTRIBUTIONS

**Stanton Nielsen**: Conceptualization; Data curation; Formal analysis; Methodology; Writing ‐ original draft; Writing ‐ review and editing. **Kristine Kuchta**: Data curation; Formal analysis; Methodology; Writing ‐ original draft; Writing ‐ review and editing. **Grace Huang**: Conceptualization; Formal analysis; Methodology; Writing ‐ original draft; Writing ‐ review and editing. **Samuel Zuber**: Conceptualization; Data curation; Formal analysis; Methodology; Writing ‐ original draft; Writing ‐ review and editing. **Simon Holoubek**: Conceptualization; Data curation; Formal analysis; Methodology; Writing ‐ original draft; Writing ‐ review and editing. **Amanda Karcioglu**: Conceptualization; Data curation; Formal analysis; Methodology; Writing ‐ original draft; Writing ‐ review and editing. **Amna Khokar**: Conceptualization; Data curation; Formal analysis; Methodology; Writing ‐ original draft; Writing ‐ review and editing. **Richard Prinz**: Conceptualization; Data curation; Formal analysis; Methodology; Writing ‐ original draft; Writing ‐ review and editing. **Tricia Moo‐Young**: Conceptualization; Data curation; Formal analysis; Methodology; Supervision; Writing ‐ original draft; Writing ‐ review and editing.

## CONFLICT OF INTEREST STATEMENT

The authors declare that they have no conflict of interest.

## ETHICS STATEMENT

The present study was conducted according to the standards set by the NorthShore University Research and Ethics Committee.

## Supporting information

Table S1

Table S2

Table S3

## Data Availability

The SEER database (SEER*STAT version 8.4.2) was queried for the PTC patients between 2004 and 2020.

## References

[1] Lim, H. , S.S. Devesa , J.A. Sosa , D. Check , and C.M. Kitahara . 2017. “Trends in Thyroid Cancer Incidence and Mortality in the United States, 1974–2013.” JAMA 317(13): 1338. 10.1001/jama.2017.2719. 28362912 PMC8216772

[2] Davies, L. , L.G.T. Morris , M. Haymart , A.Y. Chen , D. Goldenberg , J. Morris , J.B. Ogilvie , et al. 2015. “American Association of Clinical Endocrinologists and American College of Endocrinology Disease State Clinical Review: The Increasing Incidence of Thyroid Cancer.” Endocrine Practice 21(6): 686–696. 10.4158/EP14466.DSCR. 26135963 PMC4923940

[3] Krajewska, J. , A. Kukulska , M. Oczko‐Wojciechowska , A. Kotecka‐Blicharz , K. Drosik‐Rutowicz , M. Haras‐Gil , B. Jarzab , and D. Handkiewicz‐Junak . 2020. “Early Diagnosis of Low‐Risk Papillary Thyroid Cancer Results rather in Overtreatment Than a Better Survival.” Frontiers in Endocrinology 11: 571421. 10.3389/fendo.2020.571421. 33123090 PMC7573306

[4] Tufano, R.P. , S.I. Noureldine , and P. Angelos . 2015. “Incidental Thyroid Nodules and Thyroid Cancer: Considerations before Determining Management.” JAMA Otolaryngology—Head and Neck Surgery 141(6): 566. 10.1001/jamaoto.2015.0647. 25928353

[5] Burgess, J.R. , and P. Tucker . 2006. “Incidence Trends for Papillary Thyroid Carcinoma and Their Correlation with Thyroid Surgery and Thyroid Fine‐Needle Aspirate Cytology.” Thyroid 16(1): 47–53. 10.1089/thy.2006.16.47. 16487013

[6] Li, Y. , W. Che , Z. Yu , S. Zheng , S. Xie , C. Chen , M. Qiao , and J. Lyu . 2022. “The Incidence Trend of Papillary Thyroid Carcinoma in the United States during 2003–2017.” Cancer Control 29: 107327482211354. 10.1177/10732748221135447. PMC9583193 36256588

[7] Sosa, J.A. , J.W. Hanna , K.A. Robinson , and R.B. Lanman . 2013. “Increases in Thyroid Nodule Fine‐Needle Aspirations, Operations, and Diagnoses of Thyroid Cancer in the United States.” Surgery 154(6): 1420–1427. 10.1016/j.surg.2013.07.006. 24094448

[8] Ito, Y. , T. Uruno , K. Nakano , Y. Takamura , A. Miya , K. Kobayashi , T. Yokozawa , et al. 2003. “An Observation Trial without Surgical Treatment in Patients with Papillary Microcarcinoma of the Thyroid.” Thyroid 13(4): 381–387. 10.1089/105072503321669875. 12804106

[9] Ito, Y. , A. Miyauchi , H. Inoue , M. Fukushima , M. Kihara , T. Higashiyama , C. Tomoda , Y. Takamura , K. Kobayashi , and A. Miya . 2010. “An Observational Trial for Papillary Thyroid Microcarcinoma in Japanese Patients.” World Journal of Surgery 34(1): 28–35. 10.1007/s00268-009-0303-0. 20020290

[10] Kwon, H. , H.‐S. Oh , M. Kim , S. Park , M.J. Jeon , W.G. Kim , W.B. Kim , et al. 2017. “Active Surveillance for Patients with Papillary Thyroid Microcarcinoma: A Single Center’s Experience in Korea.” Journal of Clinical Endocrinology and Metabolism 102(6): 1917–1925. 10.1210/jc.2016-4026. 28323932

[11] Sugitani, I. , K. Toda , K. Yamada , N. Yamamoto , M. Ikenaga , and Y. Fujimoto . 2010. “Three Distinctly Different Kinds of Papillary Thyroid Microcarcinoma Should Be Recognized: Our Treatment Strategies and Outcomes.” World Journal of Surgery 34(6): 1222–1231. 10.1007/s00268-009-0359-x. 20066418

[12] Ito, Y. , and A. Miyauchi . 2020. “Active Surveillance of Low‐Risk Papillary Thyroid Microcarcinomas.” Gland Surgery 9(5): 1663–1673. 10.21037/gs-2019-catp-03. 33224844 PMC7667125

[13] Lohia, S. , M. Hanson , R.M. Tuttle , and L.G.T. Morris . 2020. “Active Surveillance for Patients with Very Low‐risk Thyroid Cancer.” Laryngoscope Investigative Otolaryngology 5(1): 175–182. 10.1002/lio2.356. 32128446 PMC7042648

[14] Ho, A.S. , S. Kim , C. Zalt , M.L. Melany , I.E. Chen , J. Vasquez , J. Mallen‐St. Clair , et al. 2022. “Expanded Parameters in Active Surveillance for Low‐Risk Papillary Thyroid Carcinoma: A Nonrandomized Controlled Trial.” JAMA Oncology 8(11): 1588. 10.1001/jamaoncol.2022.3875. 36107411 PMC9478884

[15] Ho, A.S. , M. Luu , C. Zalt , L.G.T. Morris , I. Chen , M. Melany , N. Ali , et al. 2019. “Mortality Risk of Nonoperative Papillary Thyroid Carcinoma: A Corollary for Active Surveillance.” Thyroid 29(10): 1409–1417. 10.1089/thy.2019.0060. 31407637 PMC7476400

[16] Haugen, B.R. , E.K. Alexander , K.C. Bible , G.M. Doherty , S.J. Mandel , Y.E. Nikiforov , F. Pacini , et al. 2016. “2015 American Thyroid Association Management Guidelines for Adult Patients with Thyroid Nodules and Differentiated Thyroid Cancer: The American Thyroid Association Guidelines Task Force on Thyroid Nodules and Differentiated Thyroid Cancer.” Thyroid 26(1): 1–133. 10.1089/thy.2015.0020. 26462967 PMC4739132

[17] Miyauchi, A. , Y. Ito , M. Fujishima , A. Miya , N. Onoda , M. Kihara , T. Higashiyama , et al. 2023. “Long‐Term Outcomes of Active Surveillance and Immediate Surgery for Adult Patients with Low‐Risk Papillary Thyroid Microcarcinoma: 30‐Year Experience.” Thyroid 33(7): 817–825. 10.1089/thy.2023.0076. 37166389 PMC10354707

[18] Tuttle, R.M. , J.A. Fagin , G. Minkowitz , R.J. Wong , B. Roman , S. Patel , B. Untch , et al. 2017. “Natural History and Tumor Volume Kinetics of Papillary Thyroid Cancers during Active Surveillance.” JAMA Otolaryngology–Head and Neck Surgery 143(10): 1015. 10.1001/jamaoto.2017.1442. 28859191 PMC5710258

[19] Molinaro, E. , M.C. Campopiano , L. Pieruzzi , A. Matrone , L. Agate , V. Bottici , D. Viola , et al. 2020. “Active Surveillance in Papillary Thyroid Microcarcinomas Is Feasible and Safe: Experience at a Single Italian Center.” Journal of Clinical Endocrinology and Metabolism 105(3): e172–e180. 10.1210/clinem/dgz113. 31652318 PMC8105780

[20] Oh, H.‐S. , J. Ha , H.I. Kim , T.H. Kim , W.G. Kim , D.‐J. Lim , T.Y. Kim , et al. 2018. “Active Surveillance of Low‐Risk Papillary Thyroid Microcarcinoma: A Multi‐Center Cohort Study in Korea.” Thyroid 28(12): 1587–1594. 10.1089/thy.2018.0263. 30226447

[21] Sanabria, A. 2018. “Active Surveillance in Thyroid Microcarcinoma in a Latin‐American Cohort.” JAMA Otolaryngology–Head and Neck Surgery 144(10): 947. 10.1001/jamaoto.2018.1663. 30178005 PMC6233831

[22] Valle, L.A. , and R.T. Kloos . 2011. “The Prevalence of Occult Medullary Thyroid Carcinoma at Autopsy.” Journal of Clinical Endocrinology and Metabolism 96(1): E109–E113. 10.1210/jc.2010-0959. 20943788

[23] Lang, W. , H. Borrusch , and L. Bauer . 1988 Jul. “Occult Carcinomas of the Thyroid. Evaluation of 1,020 Sequential Autopsies.” American Journal of Clinical Pathology 90(1): 72–76. 10.1093/ajcp/90.1.72. 3389346

[24] Russ, G. , S.J. Bonnema , M.F. Erdogan , C. Durante , R. Ngu , and L. Leenhardt . 2017. “European Thyroid Association Guidelines for Ultrasound Malignancy Risk Stratification of Thyroid Nodules in Adults: The EU‐TIRADS.” European Thyroid Journal 6(5): 225–237. 10.1159/000478927. 29167761 PMC5652895

[25] Bi, J. , P.‐F. Lyu , Y. Wang , and H. Zhang . 2023. “Survival Benefit of Active Surveillance for Papillary Thyroid Carcinoma: a Propensity Score Matching Analysis Based on SEER Database.” Frontiers in Oncology 13: 1185650. 10.3389/fonc.2023.1185650. 37361590 PMC10290187

[26] Oh, H.‐S. , H. Kwon , E. Song , M.J. Jeon , T.Y. Kim , J.H. Lee , W.B. Kim , et al. 2019. “Tumor Volume Doubling Time in Active Surveillance of Papillary Thyroid Carcinoma.” Thyroid 29(5): 642–649. 10.1089/thy.2018.0609. 30864894

[27] Tang, J. , D. Kong , Q. Cui , K. Wang , D. Zhang , X. Liao , Y. Gong , and G. Wu . 2018. “Racial Disparities of Differentiated Thyroid Carcinoma: Clinical Behavior, Treatments, and Long‐Term Outcomes.” World Journal of Surgical Oncology 16(1): 45. 10.1186/s12957-018-1340-7. 29506526 PMC5836433

[28] Bonner, A. , B. Herring , R. Wang , A. Gillis , P. Zmijewski , B. Lindeman , J. Fazendin , and H. Chen . 2023. “The Association of Socioeconomic Factors and Well‐Differentiated Thyroid Cancer.” Journal of Surgical Research 283: 973–981. 10.1016/j.jss.2022.11.033. 36915026 PMC10478758

[29] Moo‐Young, T. A. , J. Panergo , C. E. Wang , S. Patel , H. Y. Duh , D. J. Winchester , R. A. Prinz , and L. Fogelfeld . 2013. “Variations in Clinicopathologic Characteristics of Thyroid Cancer Among Racial Ethnic Groups: Analysis of a Large Public City Hospital and the SEER Database.” The American Journal of Surgery 206(5): 632–640. 10.1016/j.amjsurg.2013.07.015. 24157347

[30] Ding, J. , W. Wu , J. Fang , J. Zhao , and L. Jiang . 2020. “Male Sex Is Associated with Aggressive Behaviour and Poor Prognosis in Chinese Papillary Thyroid Carcinoma.” Scientific Reports 10(1): 4141. 10.1038/s41598-020-60199-9. 32139703 PMC7058033

[31] O’Connor, J. M. , T. Sedghi , M. Dhodapkar , M. J. Kane , and C. P. Gross . 2018. “Factors Associated with Cancer Disparities Among Low‐Medium‐And High‐Income US Counties.” JAMA Network Open 1(6): e183146. 10.1001/jamanetworkopen.2018.3146. 30646225 PMC6324449

[32] Ma, S.J. , J. Gill , O. Waldman , K. Yendamuri , C. Dunne‐Jaffe , U. Chatterjee , F. Fekrmandi , et al. 2023. “Association of Neighborhood‐Level Household Income with 21‐Gene Recurrence Score and Survival Among Patients with Estrogen Receptor–Positive Breast Cancer.” JAMA Network Open 6(2): e230179. 10.1001/jamanetworkopen.2023.0179. 36809469 PMC9945075

[33] Zhu, B. , F.‐H. Hu , Y.‐J. Jia , D.‐Y. Zhao , W.‐Q. Zhang , W. Tang , S.‐Q. Hu , et al. 2023. “Socioeconomic Status on Survival Outcomes in Patients with Colorectal Cancer: a Cross‐Sectional Study.” Journal of Cancer Research and Clinical Oncology 149(17): 15641–15655. 10.1007/s00432-023-05344-3. 37658279 PMC11797296

[34] Katsnelson, J. , R. J. Barnes , H. A. Patel , D. Monie , T. Kaufman , and N. J. Hellenthal . 2017. “Effect of Median Household Income on Surgical Approach and Survival in Renal Cell Carcinoma.” Urol Oncol Semin Orig Investig 35(9): 541.e1–541.e6. 10.1016/j.urolonc.2017.05.001. 28549821

[35] Hughes, D. T. , D. Reyes‐Gastelum , K. C. Ward , A. S. Hamilton , and M. R. Haymart . 2022. “Barriers to the Use of Active Surveillance for Thyroid Cancer Results of a Physician Survey.” Annals of Surgery 276(1): e40–e47. 10.1097/SLA.0000000000004417. 33074908 PMC8549720

[36] Schenker, Y. , A. Fernandez , R. Sudore , and D. Schillinger . 2011. “Interventions to Improve Patient Comprehension in Informed Consent for Medical and Surgical Procedures: A Systematic Review.” Medical Decision Making 31(1): 151–173. 10.1177/0272989X10364247. 20357225 PMC5419590

[37] Hsiao, V. , D.M. Elfenbein , S. C. Pitt , K.L. Long , R. S. Sippel , and D. F. Schneider . 2022. “Evaluating Discrimination of ACS‐NSQIP Surgical Risk Calculator in Thyroidectomy Patients.” Journal of Surgical Research 271: 137–144. 10.1016/j.jss.2021.10.016. 34896939 PMC8810575

[38] Abraham, C. R. , A. Ata , C. B. Carsello , T. L. Chan , S. C. Stain , and T. D. Beyer . 2014. “A NSQIP Risk Assessment for Thyroid Surgery Based on Comorbidities.” Journal of the American College of Surgeons 218(6): 1231–1237. 10.1016/j.jamcollsurg.2014.01.055. 24745620

[39] Margolick, J. , and S. M. Wiseman . 2018. “Risk of Major Complications Following Thyroidectomy and Parathyroidectomy: Utility of the NSQIP Surgical Risk Calculator.” The American Journal of Surgery 215(5): 936–941. 10.1016/j.amjsurg.2018.01.006. 29370884

[40] Zhang, L. , P. Wang , K. Li , and S. Xue . 2023. “A Novel Nomogram for Identifying High‐Risk Patients Among Active Surveillance Candidates with Papillary Thyroid Microcarcinoma.” Frontiers in Endocrinology 14: 1185327. 10.3389/fendo.2023.1185327. 37780614 PMC10541211

[41] Enewold, L. , H. Parsons , L. Zhao , D. Bott , D. R. Rivera , M. J. Barrett , B. A. Virnig , and J. L. Warren . 2020. “Updated Overview of the SEER‐Medicare Data: Enhanced Content and Applications.” Journal of the National Cancer Institute Monographs 2020(55): 3–13. 10.1093/jncimonographs/lgz029. 32412076 PMC7225666

[42] Davies, L. , and H.G. Welch . 2010. “Thyroid Cancer Survival in the United States: Observational Data from 1973 to 2005.” Archives of Otorhinolaryngology‐Head and Neck Surgery 136(5): 440. 10.1001/archoto.2010.55. 20479371

[43] Quinn, M. , and P. Babb . 2002 Jul. “Patterns and Trends in Prostate Cancer Incidence, Survival, Prevalence and Mortality. Part I: International Comparisons.” BJU International 90(2): 162–173. 10.1046/j.1464-410x.2002.2822.x. 12081758

