# Supplementary material for: Predicting disease‐specific survival in patients undergoing active surveillance for papillary thyroid carcinoma
Source: World J Surg. 2024 Dec 19;49(4):1011–21. doi: 10.1002/wjs.12434 (PMC11994147; doi:10.1002/wjs.12434)
Supplement: Supplementary file 1 — Table S1 [file WJS-49-1011-s003.docx]

| Supplemental Table 1. Survival Rates in the Training Set | | | | | | | | |
| --- | --- | --- | --- | --- | --- | --- | --- | --- |
|  |  |  |  |  | **5 Year Survival** | | **10 Year Survival** | |
|  |  | **Risk Score** | | | **Predicted** | **Observed** | **Predicted** | **Observed** |
| Vigintile | N | Mean ± SD | Min | Max | Mean | Mean | Mean | Mean |
| 1 | 5767 | 0.48 ± 0.27 | 0.00 | 0.84 | 99.99% | 99.96% | 99.98% | 99.96% |
| 2 | 6282 | 1.05 ± 0.10 | 0.84 | 1.18 | 99.99% | 99.98% | 99.97% | 99.96% |
| 3 | 5806 | 1.44 ± 0.13 | 1.18 | 1.73 | 99.98% | 100.00% | 99.95% | 99.83% |
| 4 | 6392 | 2.03 ± 0.16 | 1.74 | 2.23 | 99.96% | 99.98% | 99.91% | 99.98% |
| 5 | 5259 | 2.34 ± 0.05 | 2.24 | 2.50 | 99.94% | 99.95% | 99.87% | 99.85% |
| 6 | 6337 | 2.57 ± 0.05 | 2.50 | 2.65 | 99.93% | 99.95% | 99.84% | 99.79% |
| 7 | 5851 | 2.78 ± 0.10 | 2.65 | 2.93 | 99.91% | 99.90% | 99.80% | 99.76% |
| 8 | 5874 | 3.11 ± 0.12 | 2.93 | 3.27 | 99.88% | 99.91% | 99.73% | 99.73% |
| 9 | 6490 | 3.38 ± 0.07 | 3.27 | 3.49 | 99.84% | 99.94% | 99.64% | 99.82% |
| 10 | 5619 | 3.57 ± 0.04 | 3.49 | 3.61 | 99.81% | 99.89% | 99.57% | 99.51% |
| 11 | 5795 | 3.71 ± 0.06 | 3.62 | 3.83 | 99.78% | 99.74% | 99.50% | 99.50% |
| 12 | 6007 | 3.93 ± 0.06 | 3.83 | 4.03 | 99.73% | 99.79% | 99.38% | 99.44% |
| 13 | 5973 | 4.18 ± 0.08 | 4.04 | 4.30 | 99.65% | 99.67% | 99.20% | 99.34% |
| 14 | 6043 | 4.39 ± 0.05 | 4.30 | 4.47 | 99.57% | 99.46% | 99.02% | 98.89% |
| 15 | 5798 | 4.61 ± 0.07 | 4.48 | 4.73 | 99.46% | 99.51% | 98.78% | 98.63% |
| 16 | 5997 | 4.87 ± 0.10 | 4.73 | 5.05 | 99.30% | 99.18% | 98.42% | 98.35% |
| 17 | 5933 | 5.2 ± 0.10 | 5.05 | 5.35 | 99.02% | 98.94% | 97.81% | 97.86% |
| 18 | 6006 | 5.51 ± 0.11 | 5.35 | 5.69 | 98.67% | 98.58% | 97.01% | 96.85% |
| 19 | 5924 | 6.02 ± 0.22 | 5.69 | 6.43 | 97.76% | 97.86% | 95.01% | 95.09% |
| 20 | 5970 | 7.15 ± 0.65 | 6.44 | 10.77 | 91.69% | 92.18% | 83.04% | 84.52% |
