# Supplementary material for: Predicting disease‐specific survival in patients undergoing active surveillance for papillary thyroid carcinoma
Source: World J Surg. 2024 Dec 19;49(4):1011–21. doi: 10.1002/wjs.12434 (PMC11994147; doi:10.1002/wjs.12434)
Supplement: Supplementary file 2 — Table S2 [file WJS-49-1011-s002.docx]

| Supplemental Table 2. 10 Year Survival Rates in the Validation Set | | | | | |  |  |  |
| --- | --- | --- | --- | --- | --- | --- | --- | --- |
|  |  |  |  |  | **5 Year Survival** | | **10 Year Survival** | |
|  |  | **Risk Score** | | | **Predicted** | **Observed** | **Predicted** | **Observed** |
| Decile | N | Mean ± SD | Min | Max | Mean | Mean | Mean | Mean |
| 1 | 2980 | 0.79 ± 0.35 | -0.63 | 1.22 | 99.99% | 99.96% | 99.97% | 99.88% |
| 2 | 2995 | 1.75 ± 0.32 | 1.23 | 2.23 | 99.97% | 99.96% | 99.93% | 99.79% |
| 3 | 2925 | 2.47 ± 0.12 | 2.24 | 2.65 | 99.94% | 100.00% | 99.86% | 99.91% |
| 4 | 3019 | 2.95 ± 0.20 | 2.65 | 3.27 | 99.89% | 100.00% | 99.76% | 100.00% |
| 5 | 2978 | 3.47 ± 0.11 | 3.27 | 3.61 | 99.83% | 99.79% | 99.61% | 99.49% |
| 6 | 2978 | 3.82 ± 0.13 | 3.62 | 4.00 | 99.75% | 99.73% | 99.44% | 99.27% |
| 7 | 2972 | 4.28 ± 0.12 | 4.00 | 4.46 | 99.61% | 99.55% | 99.12% | 98.91% |
| 8 | 2958 | 4.71 ± 0.15 | 4.46 | 5.04 | 99.40% | 99.46% | 98.64% | 99.01% |
| 9 | 2995 | 5.35 ± 0.20 | 5.04 | 5.69 | 98.85% | 99.11% | 97.42% | 97.88% |
| 10 | 2981 | 6.59 ± 0.75 | 5.70 | 10.77 | 94.65% | 95.59% | 88.90% | 91.00% |
