# Supplementary material for: Predicting disease‐specific survival in patients undergoing active surveillance for papillary thyroid carcinoma
Source: World J Surg. 2024 Dec 19;49(4):1011–21. doi: 10.1002/wjs.12434 (PMC11994147; doi:10.1002/wjs.12434)
Supplement: Supplementary file 3 — Table S3 [file WJS-49-1011-s001.docx]

Supplemental Table 3. Characteristics of Patients, Active Surveillance vs. Surgery – Training Set

|  | **Active Surveillance** | **Surgery** | **p-value** |
| --- | --- | --- | --- |
|  | N (%) | N (%) | - |
| Total Patients | 1,930 | 117,193 | - |
| Year of Diagnosis |  |  | <.0001 |
| 2004 | 37 (1.9) | 4241 (3.6) |  |
| 2005 | 59 (3.1) | 4643 (4.0) |  |
| 2006 | 59 (3.1) | 5098 (4.4) |  |
| 2007 | 68 (3.5) | 5535 (4.7) |  |
| 2008 | 73 (3.8) | 6261 (5.3) |  |
| 2009 | 68 (3.5) | 6961 (5.9) |  |
| 2010 | 88 (4.6) | 6971 (5.9) |  |
| 2011 | 113 (5.9) | 7472 (6.4) |  |
| 2012 | 120 (6.2) | 7816 (6.7) |  |
| 2013 | 127 (6.6) | 8083 (6.9) |  |
| 2014 | 141 (7.3) | 8129 (6.9) |  |
| 2015 | 156 (8.1) | 8322 (7.1) |  |
| 2016 | 171 (8.9) | 7990 (6.8) |  |
| 2017 | 151 (7.8) | 7704 (6.6) |  |
| 2018 | 167 (8.7) | 7900 (6.7) |  |
| 2019 | 179 (9.3) | 7748 (6.6) |  |
| 2020 | 153 (7.9) | 6319 (5.4) |  |
| Age, years [Mean ± SD] | 56 ± 18 | 50 ± 15 | <.0001 |
| Age, years |  |  | <.0001 |
| < 30 | 128 (6.6) | 11351 (9.7) |  |
| 30-39 | 249 (12.9) | 20952 (17.9) |  |
| 40-49 | 325 (16.8) | 26410 (22.5) |  |
| 50-59 | 384 (19.9) | 27487 (23.5) |  |
| 60-69 | 355 (18.4) | 19507 (16.6) |  |
| ≥ 70 | 489 (25.3) | 11486 (9.8) |  |
| Sex |  |  | <.0001 |
| Male | 640 (33.2) | 26793 (22.9) |  |
| Female | 1290 (66.8) | 90400 (77.1) |  |
| Race |  |  | <.0001 |
| White | 1067 (55.3) | 75355 (64.3) |  |
| Black | 101 (5.2) | 6746 (5.8) |  |
| Hispanic | 396 (20.5) | 20352 (17.4) |  |
| Asian/Pacific Islander | 298 (15.4) | 13087 (11.2) |  |
| Other | 68 (3.5) | 1653 (1.4) |  |
| Median Household Income |  |  | <.0001 |
| < $60,000 | 273 (14.1) | 23616 (20.2) |  |
| $60,000-74,999 | 732 (37.9) | 41556 (35.5) |  |
| ≥ $75,000 | 925 (47.9) | 52021 (44.4) |  |
| Tumor Size, cm |  |  | <.0001 |
| 0-0.5 | 153 (7.9) | 25172 (21.5) |  |
| 0.6-1.0 | 383 (19.8) | 23368 (19.9) |  |
| 1.1-1.5 | 409 (21.2) | 21786 (18.6) |  |
| 1.6-2.0 | 284 (14.7) | 13511 (11.5) |  |
| 2.1-4.0 | 504 (26.1) | 24920 (21.3) |  |
| > 4.0 | 197 (10.2) | 8436 (7.2) |  |
| N Stage |  |  | <.0001 |
| N0 | 1354 (70.2) | 85651 (73.1) |  |
| N1 | 262 (13.6) | 27694 (23.6) |  |
| NX | 314 (16.3) | 3848 (3.3) |  |
